# First-in-human phase 1 study of the BTK inhibitor GDC-0853 in relapsed or refractory B-cell NHL and CLL

**DOI:** 10.18632/oncotarget.24310

**Published:** 2018-01-22

**Authors:** John C. Byrd, Stephen Smith, Nina Wagner-Johnston, Jeff Sharman, Andy I. Chen, Ranjana Advani, Bradley Augustson, Paula Marlton, S. Renee Commerford, Kwame Okrah, Lichuan Liu, Elaine Murray, Elicia Penuel, Ashley F. Ward, Ian W. Flinn

**Affiliations:** ^1^ Division of Hematology, Ohio State University Wexner Medical Center, Columbus, OH, USA; ^2^ Division of Medical Oncology, University of Washington, Seattle, WA, USA; ^3^ Division of Oncology, Washington University, St. Louis, MO, USA; ^4^ Willamette Valley Cancer Institute and Research Center, US Oncology, Eugene, OR, USA; ^5^ Center for Hematologic Malignancies, Oregon Health & Science University, Portland, OR, USA; ^6^ Stanford Cancer Institute, Stanford School of Medicine, Stanford, CA, USA; ^7^ Sir Charles Gairdner Hospital, Perth, WA, Australia; ^8^ Department of Haematology, Princess Alexandra Hospital, Brisbane, QLD, Australia; ^9^ Early Clinical Development, Genentech, Inc., South San Francisco, CA, USA; ^10^ Blood Cancer Research Program, Sarah Cannon Research Institute, Nashville, TN, USA

**Keywords:** CLL, GCD0853, BTK

## Abstract

GDC-0853 is a selective, reversible, and non-covalent inhibitor of Bruton’s tyrosine kinase (BTK) that does not require interaction with the Cys481 residue for activity. In this first-in-human phase 1 study we evaluated safety, tolerability, pharmacokinetics, and activity of GDC-0853 in patients with relapsed or refractory non-Hodgkin lymphoma (NHL) or chronic lymphocytic leukemia (CLL). Twenty-four patients, enrolled into 3 cohorts, including 6 patients who were positive for the C481S mutation, received GDC-0853 at 100, 200, or 400 mg once daily, orally. There were no dose limiting toxicities. GDC-0853 was well tolerated and the maximum tolerated dose (MTD) was not reached due to premature study closure. Common adverse events (AEs) in ≥ 15% of patients regardless of causality included fatigue (37%), nausea (33%), diarrhea (29%), thrombocytopenia (25%), headache (20%), and abdominal pain, cough, and dizziness (16%, each). Nine serious AEs were reported in 5 patients of whom 2 had fatal outcomes (confirmed H1N1 influenza and influenza pneumonia). A third death was due to progressive disease. Eight of 24 patients responded to GDC-0853: 1 complete response, 4 partial responses, and 3 partial responses with lymphocytosis, including 1 patient with the C481S mutation. Two additional C481S mutation patients had a decrease in size of target tumors (–23% and –44%). These data demonstrate GDC-0853 was generally well-tolerated with antitumor activity.

## INTRODUCTION

For many years, the treatment of non-Hodgkin lymphoma (NHL) and chronic lymphocytic leukemia (CLL) has involved use of chemotherapy given as monotherapy or in combination treatments. The early 1990’s brought forth the first broadly applicable non-cytotoxic monoclonal antibody therapy, rituximab, where research over the next decade would demonstrate its value across virtually all B-cell malignancies either as monotherapy or in combination with chemotherapy [1, 2]. Subsequent major breakthroughs in the development of drugs for NHL and CLL have come from the introduction of B-cell receptor antagonizing agents, such as ibrutinib, which targets Bruton’s tyrosine kinase (BTK), and idelalisib, which targets the delta isoform of phosphatidylinositol 3-kinase (PI3Kδ) [3–7]. The effectiveness of these two agents is somewhat divergent, with idelalisib demonstrating more activity in follicular lymphoma [7, 8] whereas ibrutinib has increased efficacy in mantle cell lymphoma (MCL) [9], Waldenstrom’s macroglobulinemia (WM), [10] and CLL [11, 12]. The high responses and durable remissions observed with ibrutinib in MCL, WM, and CLL are believed to be influenced by the binding properties of ibrutinib; ibrutinib covalently and irreversibly attaches to C481 in the active site of the BTK protein, thereby effectively inhibiting kinase activity until new BTK protein is generated.

BTK is a proximal kinase in B-cell receptor (BCR) signaling that amplifies activation of several down-stream factors, including PLCg2, ERK, and NFκB, which contribute to both proliferation and survival [13–15]. BTK is also involved in integrin-mediated adhesion and migration of tumor cells, and contributes to TLR9 signaling via an adaptor function of MYD88 as well [16–18]. Mouse models, which overexpress BTK through a B-cell-specific promotor, have been shown to experience increased mortality [19]. B lymphocytes from these mice are hyper-responsive to BCR stimulation and demonstrate resistance to Fas-mediated apoptosis [19]. The importance of BTK to CLL pathogenesis is best demonstrated by genetic and pharmacologic disruption of this gene in two spontaneous mouse models of this disease [20, 21]. In both, dramatic delay of development of murine CLL was observed.

With the therapeutic success of ibrutinib in CLL and other B-cell malignancies, several groups have characterized the emergence of resistance to ibrutinib therapy [22, 23]. In CLL, resistance to ibrutinib occurs either early (≤ 12 months) where Richter’s transformation is most often observed, or later (> 12 months) where CLL disease progression is common [24]. While the pathogenesis and mutational landscape of Richter’s transformation is not known, for CLL our group and others have demonstrated that mutations in the cysteine residue (C481), most often to a serine, converts ibrutinib from an irreversible covalently bound inhibitor to a reversible one with less potency [22, 25]. A follow up by our group demonstrates 80% of patients relapsing CLL will have the C481S mutation whereas 5% will have the less common PLCG2 mutation [26]. These abnormalities appear approximately 9.3 months prior to relapse [26]. For MCL, this same C481S mutation of BTK along with other mutations has been described among patients with resistance to ibrutinib [25]. For both CLL and MCL, patients developing resistance to ibrutinib is associated with rapid progression and poor outcome [24, 27, 28]. Hence, development of therapies that have the potential to both prevent disease progression and reduce development of resistance to ibrutinib is needed.

One strategy to treat C481S-mutant CLL could be the development of small molecule BTK inhibitors that do not depend upon binding to the C481 site for inhibition of BTK. Effectiveness of this approach would be reliant upon the B-cell malignancy having continued dependence on BTK. GDC-0853 is a novel BTK inhibitor that binds in a different orientation as compared to covalent inhibitors such as ibrutinib, and was designed to not require binding to the C481 site [29, 30]. GDC-0853 in preclinical models was demonstrated to have *in vitro* activity against both wild-type and C481S-mutant BTK [31]. GDC-0853-related analogs also demonstrate potency against mutant C481 BTK as well as some gatekeeper mutations [32]. Herein, we describe the first clinical use of GDC-0853 in the treatment of CLL and NHL and also extend findings observed with this treatment.

## RESULTS

### Baseline patient demographics and disease characteristics

From 17 December, 2013, to November of 2014, 24 patients were enrolled into 3 cohorts with starting doses of 100 (*n* = 6), 200 mg (*n* = 9), or 400 mg (*n* = 9) GDC-0853 (Supplementary Figure 2). In November 2014, although no MTDs had been observed, Genentech made the business decision to halt further enrollment in the study and not complete dose escalation in order to focus on the development of GDC-0853 for the treatment of autoimmune diseases including rheumatoid arthritis (NCT02983227, NCT02833350), systemic lupus erythematosus (NCT02908100), and chronic spontaneous urticaria (NCT03137069). Sites continued study execution as per protocol with the exception of enrolling 3 confirmed C481S-positive patients in the highest dose at which GDC-0853 had cleared the DLT window (i.e., 400 mg) during the following month. All other patients were allowed to continue intra-patient dose escalation up to 400 mg. This report presents data up to June 1, 2015, at which time 9 patients remained on study for duration of 271 to 467 days. All patients had undergone intra-patient dose escalation to 400 mg, the maximally assessed dose, by Jun 1, 2015.

The study enrolled 14 patients with CLL and 10 patients with NHL: 4 with follicular lymphoma (FL), 3 with diffuse large B-cell lymphoma (DLBCL), 2 with MCL, and 1 each with prolymphocytic leukemia (PLL) and Waldenström’s macroglobulinemia (WM). Patients had a median age of 68 years (range 47–81); 9 patients were over 70 years of age (38%). Patients had experienced a median of 4 prior therapies (range 2–10). Seven patients (29%) had been previously treated with a BTK inhibitor (Table 1); 6/7 of these patients were positive for the C481S mutation while 1/7 patient was not tested.

**Table 1:** Baseline demographics

Characteristic	All patients (*n* = 24)
**Age, yrs**	
median	68
range	47–81
≥ 70 yrs, *n* (%)	9 (38)
Sex, no (%)	
Female	7 (29)
Male	17 (71)
**ECOG performance status, *n* (%)**	
0	8 (33)
1	15 (63)
2	0 (0)
Not determined	1 (4)
**Histology, *n* (%)**	
CLL	14 (58)
NHL	10 (42)
FL	4
DLBCL	3
MCL	2
PLL	1
WM	1
**Number of prior systemic therapies**	
Median	4
Range	2–10
**Median time since most recent systemic anticancer therapy, months**	
Median	4
Range	1–56
**Prior BTK inhibitor treatment, n (%)**	7 (29)
**Prior XRT, *n* (%)**	6 (25)
**C481S mutation, *n* (%)**	
Positive	6 (25)
Not determined	18 (75)
**Characteristic**	**CLL patients** **(*n* = 14)**
IgVH status, *n* (%)^*^	
Not mutated (< 2%)	8 (57)
Mutated (> 2%)	3 (21)
Not determined	3 (21)
**Interphase cytogenetic abnormality, *n* (%)**	
17p deletion only	4 (29)
11q22 deletion only	4 (29)
Neither 17p nor 11q22 deletions	4 (29)
Not tested	1 (7)

^*^1 patient had both 17p and 11q deletions at study entry. 4 patients had 13q deletions and 3 patients had trisomy 12 deletions.

### Safety profile and disposition

**Figure 1:**
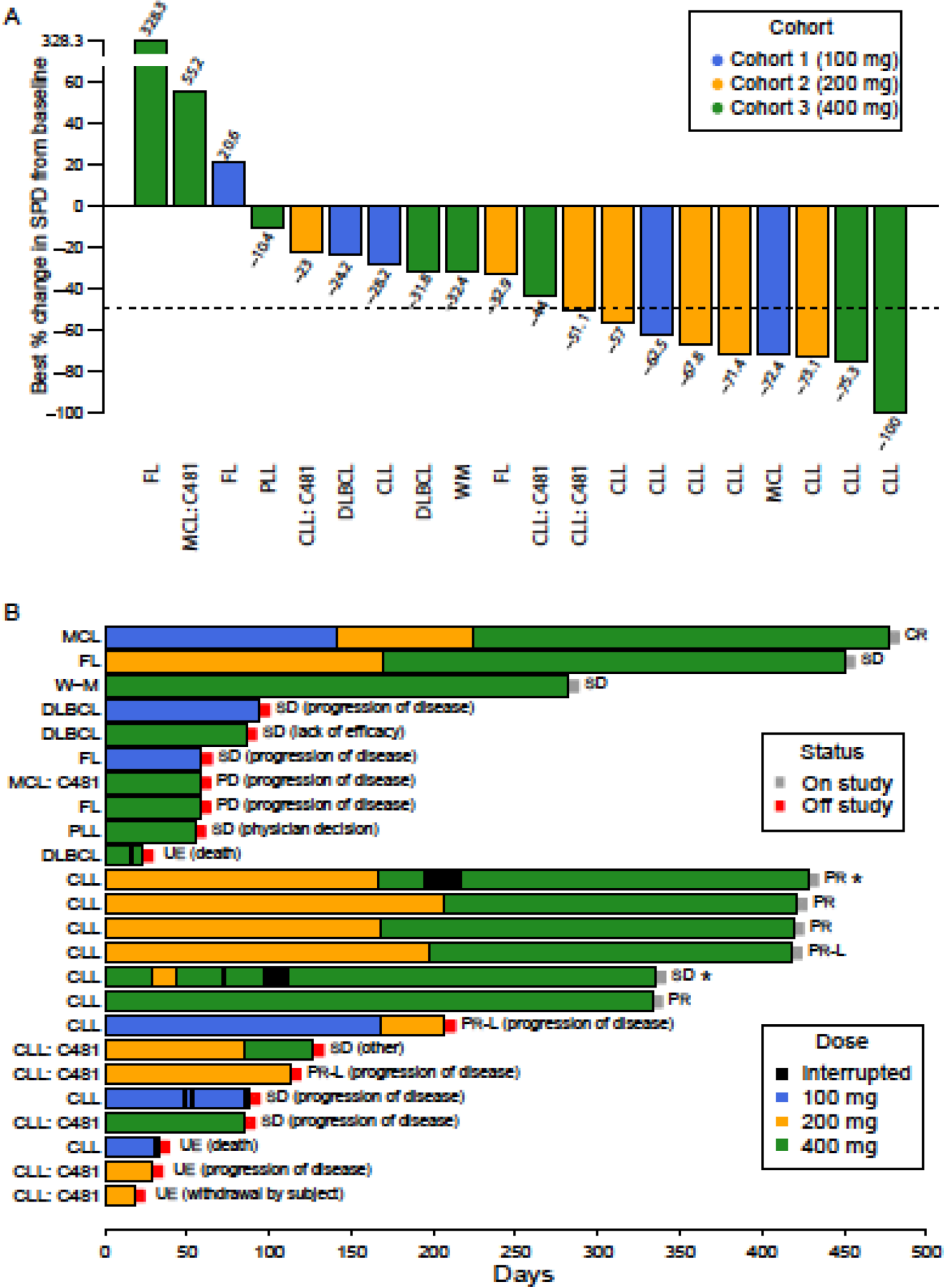
Efficacy of GDC-0853 in patients with relapsed or refractory B-cell non-Hodgkin lymphoma and chronic lymphocytic leukemia (**A**) Anti-tumor activity of GDC-0853 in target lesions. Values represent the best response of the % change from baseline of the sum of the products of the diameters (SPD) of target lymph nodes. Four patients were not evaluated for % change in SPD because of study discontinuation prior to the initial response CT evaluation. (**B**) Time on study and best overall response for all 24 patients enrolled.

At the time of data cut (Jun 1, 2015), 15 patients had discontinued treatment for the following reasons: 8 for progressive disease (PD), 3 for death, 1 for subject withdrawal, 2 for physician decision to withdraw subject; and 1 for lack of efficacy (Figure 1). No DLT’s were observed and MTD was not reached. The majority of AEs was NCI-CTCAE grade 1 or 2 and resolved without treatment or need for study drug holds. Common AEs in ≥ 15% of patients regardless of causality included fatigue (37.5%), nausea (33.3%), diarrhea (29.2%), thrombocytopenia or platelet count decreased (25.0%), headache (20.8%), and abdominal pain, cough, and dizziness (16.7%, each) (Table 2). The most common AE of grade 3 or higher was anemia, which occurred in 3 patients (12.5%). Infections of grade 3 or higher (*n* = 4) included H1N1 influenza, influenza pneumonia, lung infection, and pneumonia (1 patient each). There were 2 bleeding events of grade ≥ 3 limited to the gastrointestinal tract (gastrointestinal hemorrhage and upper gastrointestinal hemorrhage), both of which occurred in patients taking either acetylsalicylic acid (ASA) or non-steroidal anti-inflammatory drugs (NSAIDs), with known Barrett’s esophagus in 1 patient. Nine serious AEs were reported in 5 patients of whom 2 had fatal outcomes (confirmed H1N1 Influenza and influenza pneumonia, during the flu season). The reported drug related serious AEs were febrile neutropenia, lung infection, and H1N1 influenza, all of which occurred in 1 patient. AEs leading to study discontinuation occurred in 2 patients (8.33%) and included 2 of the 3 deaths during the study. The third patient death was due to progressive disease.

**Table 2:** Adverse events (AEs) by grade occurring in ≥ 15% of patients treated with GDC-0853

	Total	GDC-0853 100 mg (*n* = 6)	GDC-0853 200 mg (*n* = 9)	GDC-0853 400 mg (*n* = 9)	All Patients (*N* = 24)
**Overall**					
Number of patients with AEs		6 (100.0%)	9 (100.0%)	8 (88.9%)	23 (95.8%)
**Adverse Event**					
**Fatigue**	9 (37.5%)				
Grade 1 or 2		2 (33.3%)	3 (33.3%)	3 (33.3%)	8 (33.3%)
Grade 3 or 4		0	1 (11.1%)	0	1 (4.2%)
**Nausea**	8 (33.3%)				
Grade 1 or 2		0	4 (44.4%)	4 (44.4%)	8 (33.3%)
Grade 3 or 4		0	0	0	0
**Diarrhea**	7 (29.2%)				
Grade 1 or 2		0	4 (44.4%)	3 (33.3%)	7 (29.2%)
Grade 3 or 4		0	0	0	0
Thrombocytopenia^*^	6 (25.0%)				
Grade 1 or 2		1 (16.7%)	2 (22.2%)	1 (11.1%)	4 (16.7%)
Grade 3 or 4		2 (33.3%)	0	0	2 (8.3%)
**Headache**	5 (20.8%)				
Grade 1 or 2		1 (16.7%)	2 (22.2%)	2 (22.2%)	5 (20.8%)
Grade 3 or 4		0	0	0	0
**Dizziness**	4 (16.7%)				
Grade 1 or 2		2 (33.3%)	2 (22.3%)	0	4 (16.7%)
Grade 3 or 4		0	0	0	0
**Abdominal pain**	4 (16.7%)				
Grade 1 or 2		1 (16.7%)	1 (11.1%)	1 (11.1%)	3 (12.5%)
Grade 3 or 4		0	1 (11.1%)	0	1 (4.2%)
**Cough**	4 (16.7%)				
Grade 1 or 2		1 (16.7%)	1 (11.1%)	2 (22.2%)	4 (16.7%)
Grade 3 or 4		0	0	0	0

Percentages are based on n in the column headings. Data includes AEs with onset from the first dose of GDC-0853 through 7 days after its last dose. ^*^Thrombocytopenia includes data for the term “platelet count decreased”.

### Pharmacokinetics

**Figure 2:**
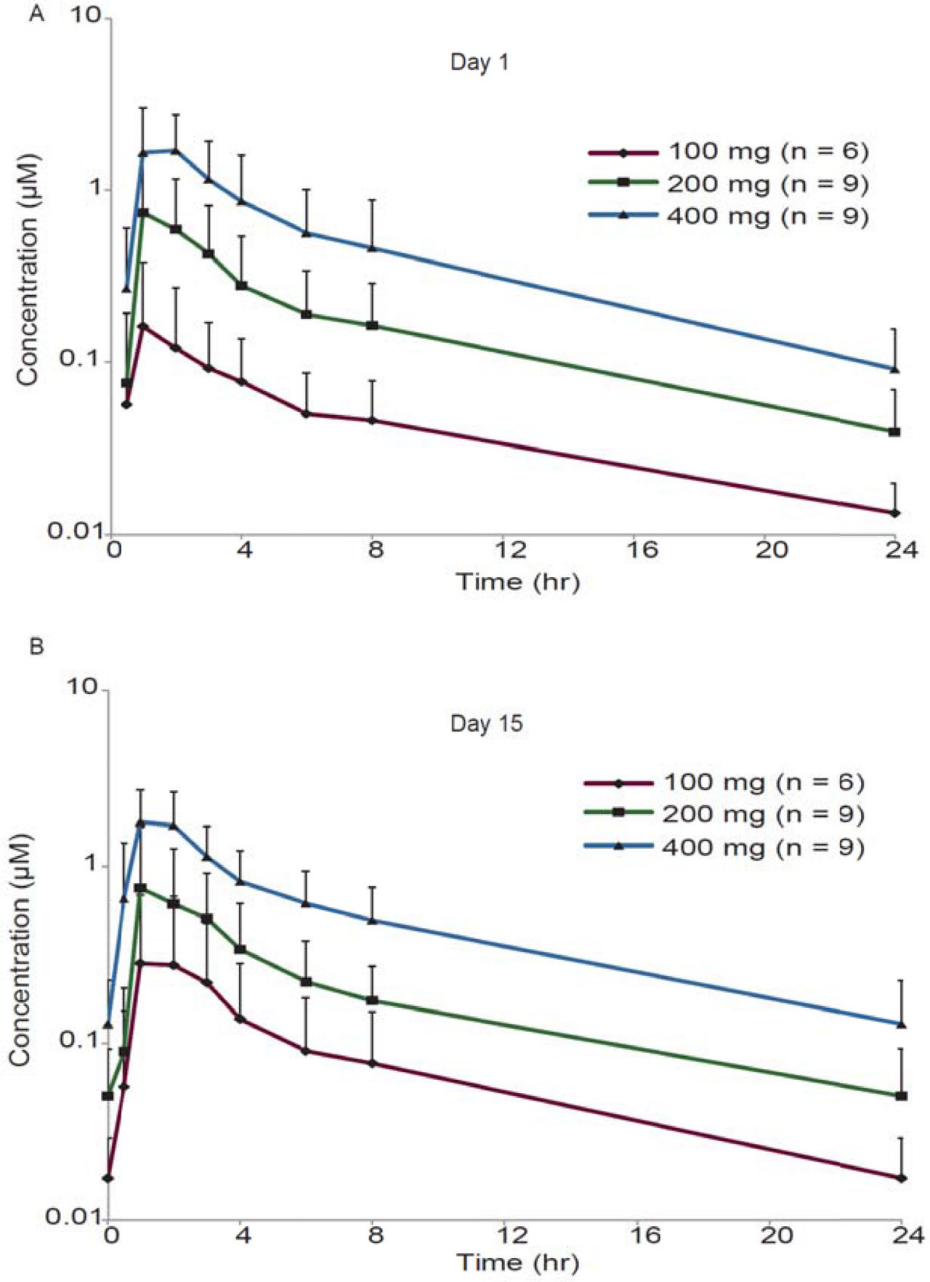
Pharmacokinetics profile of GDC-0853 Mean (±SD) GDC-0853 concentration-time profile on day 1 (**A**) and day 15 (**B**) after 100, 200, or 400 mg dose of GDC0853.

After oral administration, GDC-0853 peak concentrations (C_max_) occurred approximately 1–3 hours (median T_max_) after dosing on both days 1 and 15 (Figure 2; Table 3). Mean T_1/2_ from day 1 ranged from 6.62 to 13.7 hours. With continual QD dosing, exposure (AUC_0-24_) increase ranged from 1.44 to 1.91-fold on day 15 relative to day 1 based on the mean accumulation ratio, which suggested some accumulation of GDC-0853 after multiple doses. In general, Cmax and AUC_0-24_ increased with the dose. Overall, the variability of C_max_ and AUC in the single- and multiple-dose assessments was large, with the mean coefficient of variation (CV%) from 41.9% to 124%.

**Table 3:** Summary of pharmacokinetics parameters for GDC-0853 on day 1 and day 15 (cohorts 1, 2, and 3, with 100, 200, and 400-mg GDC-0853, respectively)

Dose	Day 1 T1/2 (hr) Mean (%CV)	Day 1 Tmaxa (hr) Median (range)	Day 1 Cmax (uM) Mean (%CV)	Day 1 AUC0-inf (hr·uM) Mean (%CV)	Day 1 AUC0-24 (hr^*^uM) Mean (%CV)	Day 15 Tmaxa (hr) Median (range)	Day 15 Cmax (uM) Mean (%CV)	Day 15 AUC0-24 (hr·uM) Mean (%CV)	Day 15 Accumulation Ratio Mean (%CV)
100 mg (*n* ***=*** 6)	13.7 (59.4)	2.07 (1.02–3.00)	0.180 (113.0)	1.30 (58.5)	1.01 (77.4)	2.97 (1.08–7.50)	0.354 (124)	1.81 (107)	1.78 (58.4)
200 mg (*n* ***=*** 9)	6.62 (41.6)	1.85 (0.833–8.03)	0.861 (90.5)	5.15 (65.2)	3.83 (76.5)	2.10 (0.917–8.00)	0.925 (106)	4.27 (63.2)	1.44 (77.9)
400 mg (*n* ***=*** 9)	7.29 (16.1)	1.17 (1.00–3.00)	2.18 (58.3)	11.4 (65.2)	10.5 (65.8)	1.05 (0.967–4.00)	2.10 (41.9)	11.7 (45.6)	1.91 (102)

AUC0-24 hr = area under the concentrationtime curve from Hour 0 to Hour 24; AUC0inf = area under the concentration time-curve from time 0 to infinity; Cmax = maximum plasma concentration; CV% = coefficient of variation; t1/2 = half-life; Tmax = time to maximum plasma concentration.

aTmax was reported as median and range.

### Plasma CCL3 concentrations

**Figure 3:**
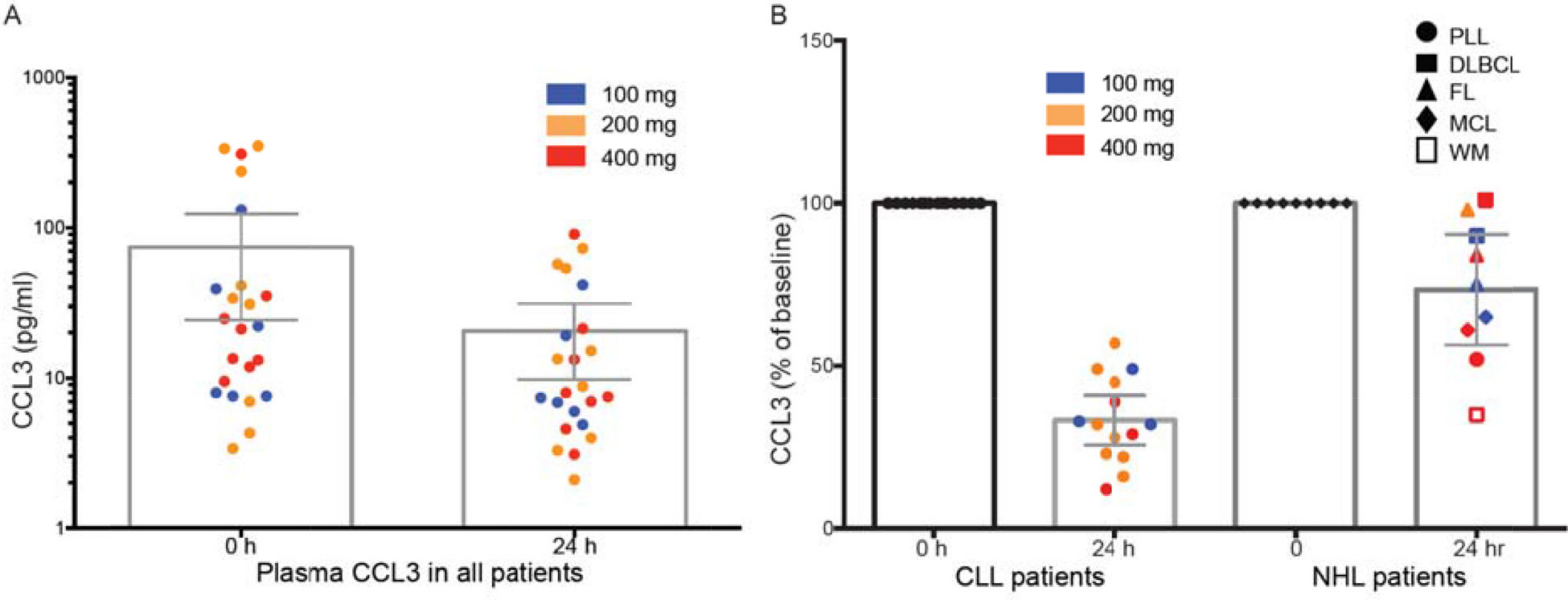
Absolute and relative CCL3 concentrations in patients treated with GDC-0853 prior to and at 24 h post first dose Error bars are 95% CI of the mean; boxes are at median. (**A**) Absolute change from baseline of plasma concentrations of CCL3 in CLL and NHL patients at baseline and 24 h after the initial oral dose of GDC-0853. (**B**) Percent change from baseline in plasma CCL3 in CLL patients or NHL patients at 24 h following an initial oral dose of GDC-0853 (100, 200, or 400 mg).

Inhibitors of BTK are thought to function by blocking BTK-dependent BCR signaling, resulting in both reduced proliferation of malignant B-cells as well as blocking of homing and chemotaxis [38]. Treatment of patients with BTK inhibitors is associated with an acute increase in peripheral blood absolute lymphocyte count and decreased blood CCL3 levels. CCL3 was explored as a pharmacodynamic marker of GDC-0853 activity. Twenty-four hours after dosing, decreased plasma CCL3 levels were observed in a majority of patients and was more apparent in those with CLL. In CLL patients, plasma CLL3 was reduced by ~67% from baseline by 24 h following the initial oral dose of GDC-0853, irrespective of starting dose (average 113 pg/ml at baseline to 28 pg/ml at 24 h) (Figure 3). In contrast, the reduction in CCL3 in NHL patients was ~-35% and ranged from −65% in 1 patient with WM to 0% in a patient with DLBCL. Importantly, CCL3 levels were reduced in patients harboring either wild-type or C481 mutations, demonstrating pharmacodynamic modulation in patients with disease that would be expected to be resistant to cysteine-481 targeted regimens.

### Clinical activity

Twenty of the 24 patients enrolled were evaluable for response to therapy. The other 4 patients discontinued before the first response assessment for disease progression, patient withdrawal, or death. Eight patients had an objective response (ORR: 8/24 in the ITT population). Best responses included 3 patients with PR-L (CLL only), 4 with PR, and 1 with CR (Table 4, Figure 3A). Of the 16 patients who were non-responders, 2 had PD at the first assessment visit (cycle 2, day 28), 10 had SD, and 4 were UE. CLL patients constituted 7 of the 8 objective responses. The single objective response from the NHL group was a CR in a patient with MCL. Overall duration of response was 3.8 ± 1.9 months (Table 4; Figure 1B; CLL: 2.5 ± 1.3 months; 10.2 months for the one NHL patient with an objective response). The 2 patients who experienced protocol violations presented with PD response outcomes at the CT assessment subsequent to their period of missed GDC-0853 doses. For both patients, the best response reported was that which occurred prior to the protocol violation (PR in one case and SD in the other, Figure 1B).

**Table 4:** Best overall responses

Histology	*n*	C481S mutation	Duration of Response (months)	Best Overall Response						
				PD	SD	PR-L	PR	CR	UE	ORR
**Overall**	24	6	3.8 ± 1.9	2	10	3	4	1	4	8/24
**CLL**	14	5	2.5 ± 1.3	0	4	3	4	0	3	7/14
**NHL**	10	1	10.2	2	6	n/a	0	1	1	1/10

CLL, chronic lymphocytic leukemia; NHL, non-Hodgkin lymphoma; PD, progression of disease; SD, stable disease; PR-L, partial response with lymphocytosis; PR, partial response; CR, complete response; UE, unevaluable, defined as those patients who were dosed with GDC-0853 but did not undergo an assessment; ORR, overall response rate; ORR reflects patients with a response of CR, PR, or PR-L and includes all patients who received study drug. Unevaluable patients are included in the ORR calculation. Duration of response is reported as median ± IQR and includes 6 patients whose data are censored, one of whom experienced a protocol violation. For this patient best overall response was that which occurred prior to the violation.

Median absolute lymphocyte count (IQR) at baseline was 5026 (15293) cells/μL (CLL) and 1330 (3175) cells/μL (NHL). As a whole, absolute lymphocytes as a median percent change from baseline increased 75 ± 169% by cycle 1, day 15 and declined towards baseline thereafter. This transient lymphocytosis was attributable largely to CLL patients (cycle 1, day 15 median increase for CLL: 120 ± 209%; for NHL: 21 ± 73%; Supplementary Table 1). Six of 14 CLL patients had baseline anemia (hemoglobin ≤ 11.0 g/dL), 1/14 had baseline neutropenia (absolute neutrophil count ≤ 1500 cells/μL), and 5/14 had baseline thrombocytopenia (absolute platelet count ≤ 100,000 cells/μL) (Supplementary Table 2). One patient with baseline anemia and 1 patient with baseline thrombocytopenia experienced sustained improvements (defined as improved cytopenia lasting for ≥ 2 consecutive cycles [11, 39]) in hemoglobin and platelet counts with daily GDC-0853 dosing (Supplementary Table 2). Interphase cytogenetics and IGVH mutational status with response data are summarized for these CLL patients. (Supplementary Table 3).

### GDC-0853 clinical activity in patients with the C481S mutation

Six patients with the C481S mutation were included in this study, 5 CLL patients and 1 patient with MCL (Table 5). In all cases, patients who had been previously treated with a BTK inhibitor (*n* = 7) and were tested for the C481S mutation were positive for mutation (1 patient who had been previously treated with a BTK inhibitor was never tested for the mutation and is therefore not included in the analysis on Table 5). Patients in this group averaged 69 years of age (range 52 to 81 years) and had undergone 7 prior therapies (range 4 to 10). Of the 6 patients, there was 1 objective response (PR-L, SPD decrease from baseline of −51.1%, response duration of 2.5 months). Two C481S patients discontinued the study prior to the first response assessment (cycle 2, day 28) and 1 patient had PD at cycle 2, day 28. Two other patients experienced SD, both of whom had some decrease in tumor size (SPD decrease from baseline of –44.0% and –23.0%) (Table 5).

**Table 5:** Characteristics and responses of patients with C481S mutations^*^

Patient no.	Histology	Age (yrs)	Prior Therapies	Best % change in SPD	Best Response^*^	Response Duration
1	MCL	81	5	55.2	PD	–
2	CLL	52	10	–51.1	PR-L	2.5 mos
3	CLL	71	6	–	UE	–
4	CLL	66	7	–23.0	SD	–
5	CLL	55	8	–	UE	–
6	CLL	73	4	–44.0	SD	–
Median	–	69	7	–33.5	–	–
Range	–	52-81	4–10	–51.1 – +55.2	–	–

MCL, mantle cell lymphoma; CLL, chronic lymphocytic leukemia; PD, progressive disease; PR-L, partial response with lymphocytosis, UE, unevaluable, defined as those patients who were dosed with GDC-0853 but did not undergo an assessment; SPD, sum of the products of the diameters.

^*^A seventh patient was previously treated with ibrutinib but was never tested for the C481S mutation. This patient’s best % change in SPD was −32% and best response was SD.

## DISCUSSION

We have described for the first time the clinical dose escalation study of GDC-0853, a novel non-covalent, reversible, selective, orally bioavailable, and ATP-competitive inhibitor of BTK that effectively blocks BCR signaling in the treatment of B-cell malignancies including CLL. Mimicking the pharmacodynamic features of covalent irreversibly-binding BTK inhibitors ibrutinib and acalabrutinib, 40 GDC-0853 caused increased blood lymphocytosis in a subset of treated CLL patients. GDC-0853 had a favorable safety profile with no dose limiting toxicity and an otherwise safe toxicity profile, including a low discontinuation rate due to toxicity and a high proportion of patients remaining on study for a prolonged period of treatment, as compared to traditional therapeutics used in this disease setting. This phase 1 trial of GDC-0853 was prematurely halted during dose escalation and the MTD was not reached. However, clinical efficacy was observed, as measured by documented partial and complete responses in CLL and MCL. Moreover, while a dose of GDC-0853 was not identified that inhibited BTK to the same continuous degree as observed with irreversible inhibitors such as ibrutinib and acalabrutinib based on pharmacodynamics measures (CCL3), 11, 40 evidence of preliminary clinical activity with this molecule was demonstrated in patients with C481S mutations following development of resistance to ibrutinib. This provides justification for continued development of reversible BTK inhibitors in CLL patients that do not depend upon the cysteine 481 binding site to inhibit BTK. While other agents such as venetoclax are active in the setting of C481S mutations but relapse inevitably occurs thereby justifying pursuit of new therapies necessary in this population

Resistance to ibrutinib in CLL and MCL has in part been shown to be mediated through acquired mutations in the C481 site, often to serine, which prevents irreversible binding and inhibition of BTK [22]. Our group has recently demonstrated that for late relapsing CLL patients, these mutations comprise the etiology of 85% of the identifiable mutations [26] Furthermore, this mutation is not pre-existing in any CLL patient prior to receiving ibrutinib, suggesting that it is acquired during treatment through as yet an unknown mechanism. CLL patients with C481S mutations in BTK have a poor outcome irrespective of treatment applied [24, 27]. Developing strategies to both treat and ideally to prevent this mutation represents a major area of research in the CLL field. Here, GDC-0853, if optimally escalated to a dose that clearly fully inhibited BTK, could serve both purposes. Even at the doses below the MTD that were administered in this trial, we demonstrate clinical activity of GDC-0853 in patients harboring C481S mutations. GDC-0853 combination regimens with ibrutinib, acalabrutinib, or other selective irreversible inhibitors could potentially be considered for patients at high risk for developing C481S mutations, such as those with complex karyotypes [24, 41].

Since GDC-0853 is a reversible inhibitor of BTK, it is not possible to employ the probe assay previously used with irreversible inhibitors to assess occupancy (e.g., ibrutinib and acalabrutinib) [11, 40]. Consequently, in the current study, CCL3 was utilized as one of the biomarkers to assess systemic inhibition of BTK in CLL. Greater inhibition of CCL3 levels were observed in the ibrutinib trials [42] as compared to this trial suggesting that further target inhibition might have been possible with further escalation of GDC-0853 or with more frequent dosing. This could explain the lower response rate observed with GDC-0853 in CLL as compared to trials with irreversible inhibitors. Importantly, unlike ibrutinib, GDC-0853 was able to inhibit BTK C481S mutants in patients, demonstrated by reductions in CCL3; dosing below the MTD may account for incomplete responses observed in these mutation-bearing patients. More definitive conclusions may have been derived from this study had dose escalation with GDC-0853 been completed and represents a major limitation of this study. However, the findings from this trial provide rationale for study of other such reversible inhibitors in this subset of patients with C481S mutations.

## MATERIALS AND METHODS

### Study design

The primary objective of this first-in-human phase 1, open-label, dose-escalation study was to evaluate the safety and tolerability of GDC-0853 administered orally to patients with relapsed or refractory NHL or CLL, and to characterize the maximum tolerated dose (MTD) and dose-limiting toxicities (DLTs) of GDC-0853. The study was designed as a standard 3 + 3 dose escalation trial that included intra-patient dose-escalation and a planned expansion cohort in several CLL and NHL sub-groups at the recommended phase 2 dose (RP2D). DLTs were pre-defined as the following events occurring during cycle 1 (days 1–35): any treatment-related grade ≥ 3 non-hematological adverse events (AEs) with the exception of alopecia of any grade and grade ≥ 3 nausea, vomiting, or diarrhea that responds to treatment within 3 days, grade 4 thrombocytopenia or grade 3 thrombocytopenia with clinically significant bleeding, grade 4 neutropenia lasting ≥ 5 days, febrile neutropenia, or grade 4 anemia. The MTD was defined as the dose at which ≤ 2 out of 6 patients at an assigned dose presented with a protocol-defined DLT. Pharmacokinetics (PK) of GDC-0853 were also assessed.

The study was conducted at 9 sites, 7 in the US and 2 in Australia. The protocol was approved by Institutional Review Boards prior to patient recruitment and was conducted in accordance with International Conference on Harmonization (ICH) E6 Guidelines for Good Clinical Practice. All patients provided written informed consent. This trial was registered at ClinicalTrials.gov (NCT01991184). Data were analyzed centrally at Genentech; all authors had access to primary clinical trial data.

Following screening and consent, patients received a single dose of GDC-0853 for PK evaluation on day 1 at their assigned dose level to assess single dose PK. Seven days later (day 8), 4 weeks of continuous dosing were implemented (Supplementary Figure 1), during which time steady-state PK was assessed. All successive cycles were 28 days in length. After completion of cycle 1 (day 35) and in the absence of unacceptable toxicity or disease progression, patients received continued treatment with GDC-0853 in consecutive 28-day cycles (4 weeks of continuous daily dosing). Intra-patient dose escalation was allowed only in patients who did not experience a drug-related AE that met the criteria for a DLT and had completed two cycles of GDC-0853 at the currently assigned dose.

### Patients

Patients were enrolled on this study with histologically documented, incurable B-cell hematologic malignancy (NHL or CLL) that had progressed despite standard of care therapy and for which there was no alternative therapy of proven benefit. Other inclusion criteria were age ≥ 18 years, Eastern Cooperative Oncology Group (ECOG) performance status of 0–1, measurable disease (defined by CT scan), tumor specimen or peripheral blood availability (CLL only) and adequate hematologic function (neutrophil count > 1000/μL, hemoglobin > 9 g/dl, and platelets > 75,000/μL) if marrow disease was not present. Additionally, patients were required to have a total bilirubin ≤ 1.5 × upper limit of normal (ULN), AST and ALT ≤ 3 × ULN, Serum creatinine ≤ 1.5 × ULN, international normalized ratio (INR) ≤ 1.5 × ULN, and activated partial thromboplastin time (aPTT) ≤ 1.5 × ULN. All patients met clinical indication for treatment as determined by the investigator. Major exclusion criteria included life expectancy of < 12 weeks, < 3 weeks since last anti-tumor therapy, surgical procedure within 4 weeks of treatment, active infection requiring intravenous treatment, human immunodeficiency virus infection, history of vasculitis or pancreatitis within 5 years, history of stroke or intracranial hemorrhage within 6 months, or history of CNS malignancy. Screening laboratory assessments were performed locally. Testing of interphase cytogenetic abnormalities in CLL patients was performed at a central laboratory. C481S mutation testing was performed locally as previously described [24].

### Study assessments

AEs were graded using the National Cancer Institute Common Terminology Criteria for AEs (NCICTCAE), version 4.03 (https://evs.nci.nih.gov/.../CTCAE/CTCAE_4.03_2010-06-14_QuickReference_5×7.pdf). On the first day of every cycle, patients underwent a targeted physical examination, confirmation of concomitant medications, assessment of vital signs, B symptoms (> 10% weight loss, fever of > 38°C, and/or night sweats), ECOG performance status, AEs and laboratory testing for hematology, clinical chemistry, and urinalysis. Disease status was assessed at screening and after every even numbered cycle for cycles 1-6 (starting with cycle 2), then after every third cycle starting with cycle 9. Two protocol violations occurred during this study, one related to an AE and the other to an error in medication; the best response reported for the two patients is that which occurred prior to the protocol violation.

### Pharmacokinetics and plasma biomarkers

PK parameters were calculated on study days 1 and 15 based on the plasma concentration of GDC-0853 according to the model independent approach. PK (plasma) samples were collected at predose, 0.5, 1, 2, 3, 4, 6, 8, and 24 h post-dose, and analyzed at a sponsor-designated laboratory using a validated liquid chromatography–tandem mass spectrometry (LC-MS/MS) method. CCL3 was determined in plasma samples using the Erenna Immunoassay System (Singulex Inc, Alameda, CA) with a fluorescently labeled CCL3 detection antibody (P&D System; AF270-NA) [33, 34]. Mutational assessment of BTK mutation status was performed according to methods previously reported [22]. Positivity was considered when 3% or more of the cells derived from blood CD19 selected B-cells were positive for C481S mutation in a CLIA approved laboratory.

### Statistical analysis

The data cut-off date for all analyses was Jun 1, 2015. All patients who received at least 1 dose of GDC-0853 were included in both safety and clinical activity analyses. Preliminary assessment of anti-tumor activity of GDC-0853 was a secondary objective of the study. Patients who were unevaluable (UE) (i.e. those patients who did not complete a response assessment) were categorized as non-responders. The overall response rate (ORR) was determined as responder patients (complete response [CR] and partial response [PR], or partial response with lymphocytosis [PR-L] for CLL patients) relative to all patients in the study. Duration of objective response was calculated as the time from the first occurrence of a documented objective response until the time of relapse, death from any cause, or the date of the last tumor assessment prior to the data cut-off (censored). Anti-tumor activity of GDC-0853 was determined by the change in the sum of the products of the diameters (SPD) of the target lesions relative to baseline, estimated by computed tomography (CT) scans. Response for CLL was determined using the IWCLL guidelines [35]. and also included PR-L as described by Cheson et al. [36] For NHL, response was based on physical examinations and CT scans using modified response criteria for NHL [37]. PK concentrations are expressed as mean ± standard deviation. Hematology (neutrophils, platelets, lymphocytes, and hemoglobin) and plasma analytes are presented either as absolute concentrations (median ± interquartile range or mean ± 95% CI) or as a percent change from baseline (visit cycle 1, day 1). Response duration is reported as median ± IQR.

## SUPPLEMENTARY MATERIALS FIGURES AND TABLES


