# Correction: First-in-human phase 1 study of the BTK inhibitor GDC-0853 in relapsed or refractory B-cell NHL and CLL

**DOI:** 10.18632/oncotarget.27011

**Published:** 2019-06-04

**Authors:** John C. Byrd, Stephen Smith, Nina Wagner-Johnston, Jeff Sharman, Andy I. Chen, Ranjana Advani, Bradley Augustson, Paula Marlton, S. Renee Commerford, Kwame Okrah, Lichuan Liu, Elaine Murray, Elicia Penuel, Ashley F. Ward, Ian W. Flinn

**Affiliations:** ^1^ Division of Hematology, Ohio State University Wexner Medical Center, Columbus, OH, USA; ^2^ Division of Medical Oncology, University of Washington, Seattle, WA, USA; ^3^ Division of Oncology, Washington University, St. Louis, MO, USA; ^4^ Willamette Valley Cancer Institute and Research Center, US Oncology, Eugene, OR, USA; ^5^ Center for Hematologic Malignancies, Oregon Health & Science University, Portland, OR, USA; ^6^ Stanford Cancer Institute, Stanford School of Medicine, Stanford, CA, USA; ^7^ Sir Charles Gairdner Hospital, Perth, WA, Australia; ^8^ Department of Haematology, Princess Alexandra Hospital, Brisbane, QLD, Australia; ^9^ Early Clinical Development, Genentech, Inc., South San Francisco, CA, USA; ^10^ Blood Cancer Research Program, Sarah Cannon Research Institute, Nashville, TN, USA

**This article has been corrected:** Due to errors in figure preparation, the data for Table 3 was listed incorrectly. It has now been updated with corrected numbers (see text in Table 3) and the resulting graphs (Figure 2A and 2B) have been updated as well. In addition, the reference list for this paper was accidentally alphabetized during the production process, while citation numbers within the article remained according to first-mention. In this correction, the entire references list has been corrected. We include the reference list in their order of mention so that the citation number in the article corresponds to the reference number in the list.

Original article: Oncotarget. 2018; 9:13029–13035. 13023-13035 . https://doi.org/10.18632/oncotarget.24310

**Table 3:** Summary of pharmacokinetics parameters for GDC-0853 on day 1 and day 15 (cohorts 1, 2, and 3, with 100, 200, and 400-mg GDC-0853, respectively)

Dose	Day 1 T_1/2_ (hr) Mean (%CV)	Day 1 T_max_^a^ (hr) Median (range)	Day 1 C_max_ (μM) Mean (%CV)	Day 1 AUC_0-inf_ (hr•μM) Mean (%CV)	Day 1 AUC_0-24_ (hr*μM) Mean (%CV)	Day 15 T_max_^a^(hr) Median (range)	Day 15 C_max_ (μM) Mean (%CV)	Day 15 AUC_0- 24_ (hr•μM) Mean (%CV)	Day 15 Accumulation Ratio Mean (%CV)
100 mg (*n* = 6)	13.7 (59.4)	2.07 (1.02–3.00)	0.119 (113.0)	0.861 (58.5)	0.670 (77.4)	2.97 (1.08–7.50)	0.235 (124)	1.20 (107)	1.78 (58.4)
200 mg (*n* = 9)	6.62 (41.6)	1.85 (0.833– 8.03)	0.571 (90.5)	3.42 (65.2)	2.54 (76.5)	2.10 (0.917– 8.00)	0.614 (106)	2.83 (63.2)	1.44 (77.9)
400 mg (*n* = 9)	7.29 (16.1)	1.17 (1.00–3.00)	1.44 (58.3)	7.57 (65.2)	6.95 (65.8)	1.05 (0.967– 4.00)	1.39 (41.9)	7.74 (45.6)	1.91 (102)

AUC_0-24hr_ = area under the concentration time curve from Hour 0 to Hour 24;

CAUC_0inf_ = area under the concentration time-curve from time 0 to infinity;

C_max_ = maximum plasma concentration; CV% = coefficient of variation; t_1/2_ = half-life;

T_max_ = time to maximum plasma concentration.

aT_max_ was reported as median and range.

**Figure 2:**
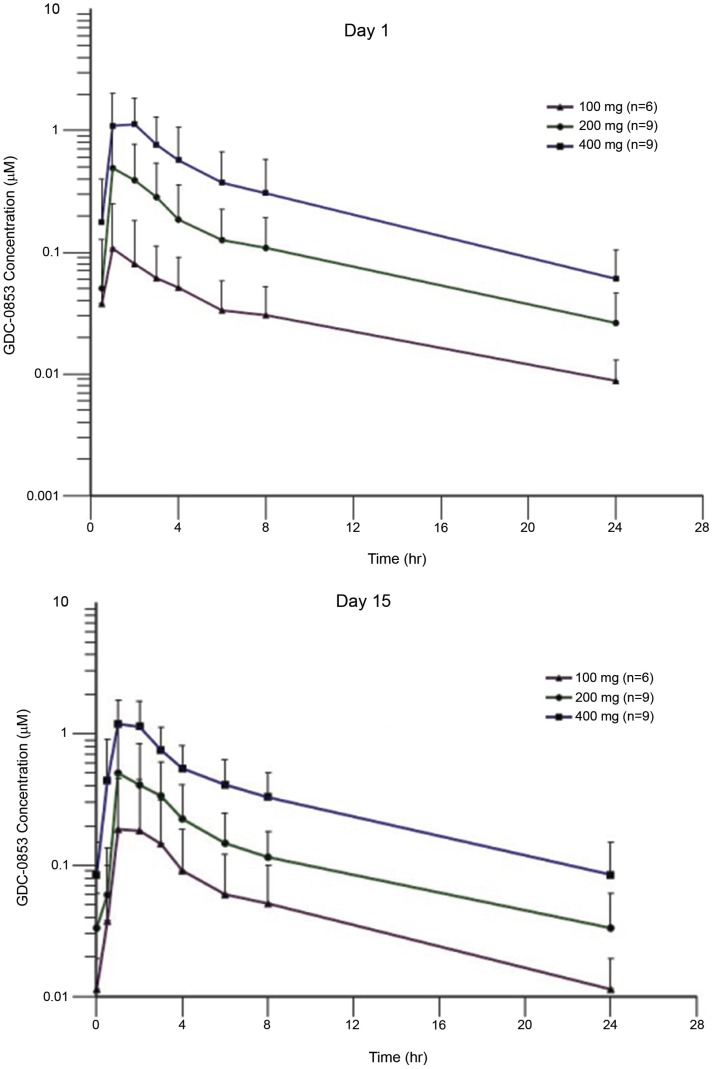
Pharmacokinetics profile of GDC-0853. Mean (±SD) GDC-0853 concentration-time profile on day 1 **(A)** and day 15 **(B)** after 100, 200, or 400 mg dose of GDC0853.
