# Supplementary material for: First-in-human phase 1 study of the BTK inhibitor GDC-0853 in relapsed or refractory B-cell NHL and CLL
Source: Oncotarget. 2018 Jan 22;9(16):13023–35. doi: 10.18632/oncotarget.24310 (PMC5849192; doi:10.18632/oncotarget.24310)
Supplement: Supplementary file 1 [file oncotarget-09-13023-s001.pdf]

First-in-human phase 1 study of the BTK inhibitor GDC-0853 in relapsed or refractory B-cell NHL and CLL

SUPPLEMENTARY MATERIALS

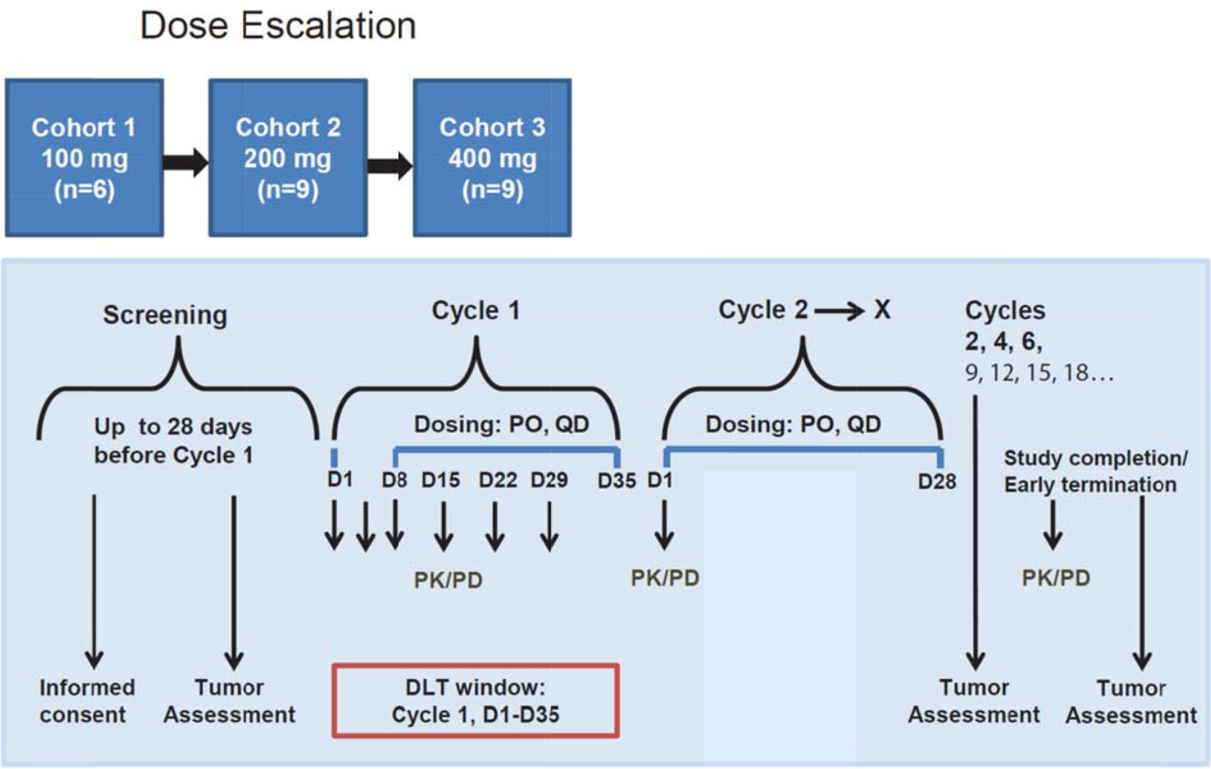

Supplementary Figure 1: Study scheme. Patients underwent screening activities, including tumor assessment by computed tomography (CT), prior to study enrollment. On day 1 of cycle 1 (C1D1), patients took a single oral dose of GDC0853 and underwent periodic blood draws over the next 24 h to characterize the acute PK of the drug. Following a 7-day washout, patients began daily oral dosing of GDC0853 on day 8 with assessments as described in METHODS on days 8, 15, 22, 29, and 35. Day 35 of the study represented the end of the DLT window (35 days). Once a patient cleared the DLT window, they continued daily PO dosing with GDC-0853. Blood assessments occurred at the end of every cycle (28 days); tumor size was assessed by CT at the end of every even-numbered cycle through cycle 6, and then every third cycle thereafeter. At the time of study completion/early termination, PK and tumor assessments were repeated.

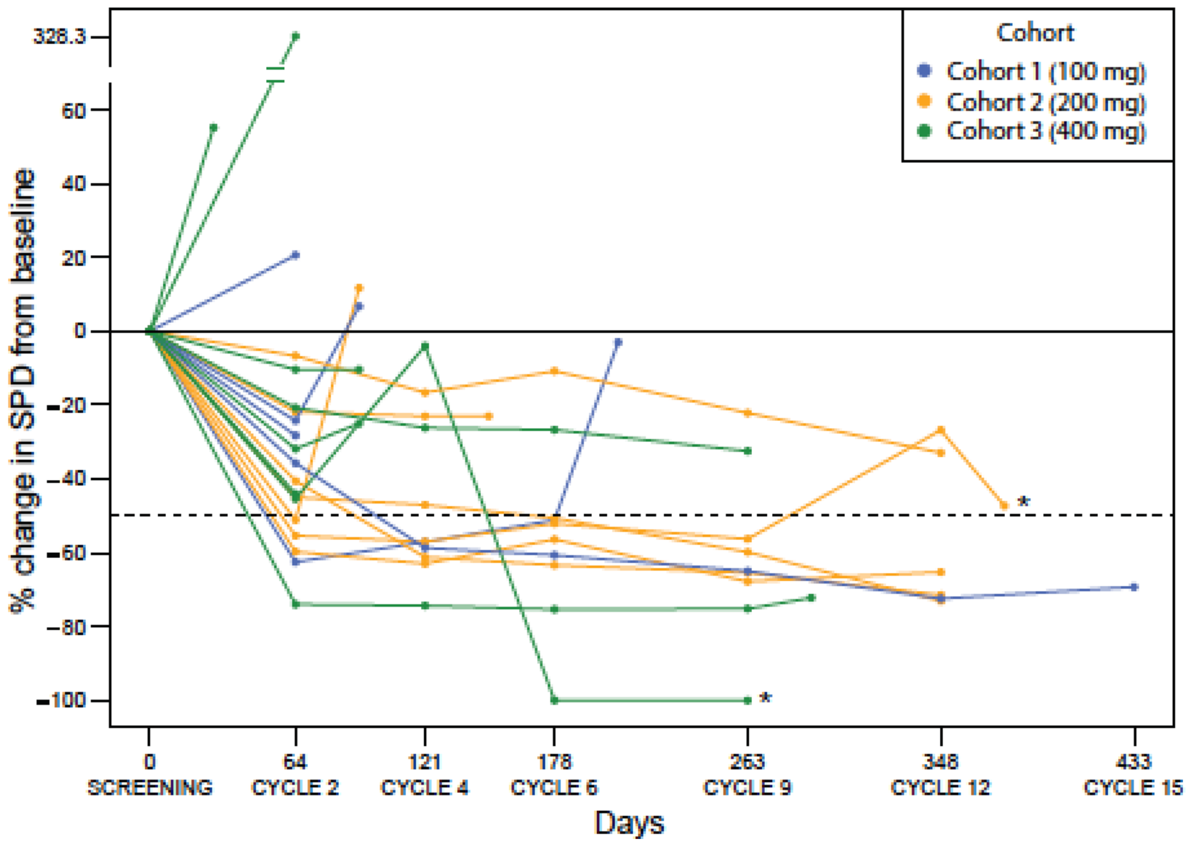

Supplementary Figure 2: Anti-tumor activity of GDC-0853 in target lesions for each patient over time. Values represent the percent change from baseline of the sum of the product of the perpendicular diameters (SPD) of target lesions. Colors represent the starting dose for each patient even though patient may have undergone dose escalation. Two protocol violations (*) are described in METHODS.

Supplementary Table 1: Changes in lymphocytes with GDC-0853 as absolute count and percent change from baseline

	C1D1 (baseline)	C1D8	C1D15	C1D22	C1D29	C2D1	C2D15	C3D1
**All patients**								
• ALC	3575	3635	9440	8404	10690	14000	11050	20228
• (IQR)	(5022)	(18382)	(41979)	(20770)	(33525	(22465)	(19530)	(22298)
• % change (IQR)	0	9 (35)	75 (169)	59 (156)	38 (203)	36 (283)	39 (141)	19 (370)
• *N*	24	24	24	24	23	21	21	16
**CLL patients**								
• ALC	5026	5245	13125	11690	13400	18725	20480	22130
• (IQR)	(15293)	(28642)	(46245)	(30775)	(33900)	(19771)	(14737)	(10400)
• % change (IQR)	0	3 (28)	120 (209)	136 (279)	103 (275)	116 (411)	120 (416)	56 (517)
• *N*	14	14	14	14	13	12	12	11
**NHL patients**								
• ALC	1330	1416	1770	1825	1845	1700	1950	2240
• (IQR)	(3175)	(5970)	(10912)	(6604)	(7612)	(7745)	(1009)	(2302)
• % change (IQR)	0	14(34)	21 (73)	16 (90)	32 (87)	25 (61)	13 (74)	−15 (23)
• *N*	10	10	10	10	10	9	9	5
**C481S mutation**								
• ALC	7265	22750	31095	9740	10690	16615	15670	12900
• (IQR)	(15312)	(47567)	(44872)	(25397)	(33900)	(18648)	(14347)	(10365)
• % change (IQR)	0	30 (114)	159 (332)	132 (140)	74 (67)	27 (101)	59 (67)	−3 (36)
• *N*	6	6	6	6	5	4	4	3

ALC, absolute lymphocyte count in cells/uL. Percent change is calculated from the C1D1 baseline value. Values represent the median ± interquartile range (IQR) of the percent change from baseline at visit Cycle 1, Day 1. CLL, chronic lymphocytic leukemia; NHL, non-Hodgkin's lymphoma.

Supplementary Table 2: Changes in cytopenias in CLL patients treated with GDC-0853

**Anemia**	
Number of CLL patients with baseline hemoglobin ≤ 11.0 g/dL	6/14
Number of CLL patients with sustained* on-treatment hemoglobin > 11.0 g/dL*	1/6
**Neutropenia**	
Number of CLL patients with baseline ANC ≤ 1500 cells/µL	1/14
Number of CLL patients with sustained* on-treatment ANC > 1500 cells/µL*	0/1
**Thrombocytopenia**	
Number of CLL patients with baseline platelets ≤ 100,000 cell/µL	5/14
Number of CLL patients with sustained* on-treatment platelets > 100,000 cell/µL*	1/5

*Sustained on-treatment is defined as maintenance of hemoglobin > 11.0 g/dL (anemia), ANC (absolute neutrophil count) >1500 cell/µL (neutropenia), or absolute platelet count > 100,000 cells/µL (thrombocytopenia) for ≥ 2 cycles in patients who entered the study with baseline cytopenia.

Supplementary Table 3: Prognostic factors for CLL* outcome

Gene	Total	Responders	Nonresponder
**13q deletion**			
Negative	8	5	3
Positive	5	3	2
**11q22 deletion**			
Negative	8	4	4
Positive	5	4	1
**CEN12 deletion**			
Negative	10	7	3
Positive	3	1	2
**p53(17p) deletion**			
Negative	8	5	3
Positive	5	3	2
**IGVH mutated < 2%**			
Yes	8	4	4
No	3	2	1

*8 CLL patients were responders (best response of CR, PR, or PR-L) and 6 were non-responders (3 SD and 3 UE).
